# An Assessment of the Relationship between Anthropometric Parameters and Blood Pressure among Polokwane Private School Children

**DOI:** 10.3390/children7040029

**Published:** 2020-04-03

**Authors:** Betty Sebati, Kotsedi Monyeki, Phuti Makgae

**Affiliations:** Department of Physiology and Environmental Health, University of Limpopo, Polokwane, Sovenga 0727, South Africa; bettysebati800@gmail.com (B.S.); pjmakgae@yahoo.com (P.M.)

**Keywords:** blood pressure, anthropometry, children, association, body composition

## Abstract

High blood pressure (HBP) among children and adolescents has been associated with elevated risk of cardiovascular diseases later in life. The aim of this study was to determine the relationship between body composition and blood pressure among Polokwane private school children. Mean body fat % was significantly (P < 0.05) higher in girls (23.74) than the boys (16.77). There was a significant (P < 0.05) association between systolic blood pressure (BP) and waist circumference (WC) unadjusted (OR = 1.125) and adjusted (OR = 1.097) for age and gender. This study included a total of 1665 children and adolescents (846 boys and 819 girls) aged 5 to 15 years old. Anthropometric measurements including weight, height, hip circumference (HC) and waist circumference (WC) were taken according to standard procedures. Descriptive statistics were done to determine the prevalence of hypertension and mean of all the variables. Pearson correlation, linear regression and logistic regression were all done to determine the association between blood pressure (BP) and the anthropometric measurements. All statistical analysis were done using SPSS. There was a significant association between body composition and blood pressure among Polokwane Private School children. Lowering the risk factors of high BP in children and adolescents will lower their risk of cardiovascular diseases in adulthood.

## 1. Introduction

High blood pressure (HBP) among children and adolescents has been associated with elevated risk of cardiovascular diseases later in life [1]. It has also been reported to cause the early development of pathological lacerations of atherosclerosis [2]. Increase in weight is related to increments in HBP, which could also lead to atherosclerotic disease later in life [3,4]. Moreover, fat is associated with several health problems, including cardiovascular disease and metabolic, pulmonary, neurological, orthopaedic and social disorders [3]. Several other factors have an impact on blood pressure, including gender, age as well as body composition (BC) [5]. Therefore, it is vital that high blood pressure monitoring begin early in childhood.

Anthropometric measurements including BMI, waist circumference (WC), and skinfold thickness, are a noninvasive and affordable technique to evaluate children’s nutritional status and have been recommended for wide use in clinical practice [2]. There are some uncertainties with regard to the determinants of blood pressure in childhood. Some studies have reported a positive association between BP and body mass index (BMI) (used to measure overall obesity) in children [6,7]. However, BMI has been contended as a crude measure of adiposity and has been reported to be poor in distinguishing between fat and lean muscle [8]. The correlations that were found between BMI and body fat contrast greatly among studies, and that puts further doubts into their association [5]. It has also been observed that BMI measurements in childhood may be complicated by growth and development [8]. The measurement of abdominal fat in addition to obesity has been extensively used to advance the assessment of cardiometabolic risk [9]. This is because the fat pattern distribution greatly influences cardiometabolic risk [10]. Consequently, abdominal obesity appears to be a better predictor for the development of cardiovascular diseases than overall obesity [11]. The waist circumference (WC) and the waist-to-hip ratio (WHR) are the broadly recognized measures for assessing abdominal obesity [12].

Numerous studies have reported the prevalence of HBP among children in developed areas in and out of South Africa [2,13] which ranged from 4.8% to 5.3% in children as little as 9 years old in South Africa. Furthermore, no studies have reported about the relationship between blood pressure and body composition in Limpopo Province to the best of my knowledge. Therefore, the aim of this study was to determine the relationship between blood pressure and body composition among Polokwane Private School children.

## 2. Materials and Methods

### 2.1. Sample and Setting

This study included a total of 1665 children aged 5 to 15 years from Polokwane Private School (846 boys mean age 10.02 years, and 819 girls mean age 9.73). Most of the participants were black children (99.77%) and 0.2% were white; 0.01% were colored and 0.02% were Indians, which resulted in their exclusion from the analysis. The study took part in Polokwane, a city in Limpopo Province, South Africa. Children who were available at the schools during the days of the survey participated in the study.

The ethics committee of the University of Limpopo granted ethical approval prior to the study (ethical clearance number MREC /P/204/2013: IR). Written informed consent was attained from parents and guardians of the children.

### 2.2. Anthropometric Measurements

All participants undertook a sequence of anthropometric measurements of waist circumference (WC), weight and height following standard procedures of the International Society for the Advancement of Kinanthropometry [14]. Trained research assistants provided the needed support during data collection. Skinfolds (subscapular and triceps) were measured three times using a Slim Guide skinfold calliper. An automated scale was utilized to carry out weight measurements (to the closest 0.1 kg), while height was measured using a Martin anthropometer (to the closest 0.1 cm). Body mass index (BMI) was subsequently calculated based on the participants’ weight and height measurements. Waist circumference (WC) and hip circumference (HC) were measured to the nearest 0.1 cm using a retractable steel tape measure. Measurements of WC were undertaken as the participants stood upright and after a mild expiration. The anatomical landmarks used for WC were, laterally, the midpoint between the iliac crest and the bottom part of the thoracic cage, and anteriorly, the midpoint between the xiphoid process of the navel and sternum, while for HC the tape was placed in a horizontal plane around the hips at the point of greatest circumference. Waist-to-hip ratio (WHR) was calculated by dividing the WC by HC [14].

Percentage body fat was based on the sum of triceps and subscapular skinfolds using the equation below [15,16]:

Boys (all ages) body fat % = 1.21 (triceps + subscapular)
−0.008 × (triceps +subscapular) 2 +1.7
  Girls (all ages) body fat %: 1.33 × (triceps + subscapular)
−0.013 × (triceps + subscapular) 2 − 2.5
For participants with a sum of triceps and subscapular <35 mm.

Then the following equation was used:

Boys (all ages) body fat % = 0.783 (triceps + subscapular) + 1.6
Girls (all ages) body fat %=0.546 (triceps + subscapular) + 9.7

### 2.3. Blood Pressure

Using an electronic Micronta monitoring kit, at least three readings of systolic blood pressure (SBP) and diastolic pressure (DBP) were taken at an interval of five minutes apart after the participants had been seated for 5 minutes or longer [17], the average from the three BP readings were calculated.

Blood pressure measurements were categorised according to SBP and/or DBP percentiles as follows: normal BP—average SBP and/or average DBP <90th percentile; prehypertension—average SBP and/or average DBP ≥90th percentile but <95th percentile; and hypertension—average SBP and/or average DBP ≥95th percentile [2].

### 2.4. Statistical Analysis

Descriptive statistics (mean and standard deviation) were done for age, blood pressure and all the body composition variables (weight, height, BMI, WC, HC, WHR, triceps, subscapular, body fat %), while descriptive frequencies were done for blood pressure only. Pearson correlation coefficients were performed to determine the relationship between blood pressure and the body composition variables by gender. Linear regression was also performed to determine the relationship between blood pressure and the body composition variables by gender. Logistic regression was performed to further determine the relationship between blood pressure and the body composition variables. All statistical analysis was done using SPSS version 25 and the P value was set at P < 0.05.

## 3. Results

Table 1 shows the physiological and anthropometric characteristics of Polokwane Private School children by gender. Boys had a significantly (P < 0.05) higher mean age (10.02 years) than the girls (9.73 years), while girls had a significantly (P < 0.05) higher mean HC (72.53 cm) than males (68.70 cm).There was no significant difference (P > 0.05) between the mean for the height of boys (139.17 cm) and girls (139.38 cm). Moreover, the mean body fat % was significantly (P < 0.05) higher in girls (23.74) than the boys (16.77).

Table 2 shows the descriptive frequencies of systolic and diastolic blood pressure among Polokwane Private School children by gender. There was a higher prevalence of both high systolic and high diastolic blood pressure in females (5.7% and 6.1%) than males (4.8% and 5.1%), respectively. The prevalence of hypertension was also higher in females (5.3%) than males (3.3%).

Table 3 shows Pearson correlation between systolic and diastolic blood pressure and the physiological and anthropometric characteristics of Polokwane Private School children by gender. There was a significant (P < 0.001) correlation between systolic BP and all the physiological and anthropometric characteristics of the participants including BMI (r = 0.438), triceps (r = 0.330), body fat % (r = 0.310) and WC (r = 0.484) in all the participants. Furthermore, there was a significant (P < 0.001) correlation between diastolic BP and all the physiological and anthropometric characteristics of the participants such as weight (r = 0.574); HC (0.546) and age (r = 0.481).

Table 4 shows linear regression showing the relationship between blood pressure and the physiological and anthropometric characteristics of Polokwane Private School children by gender. There was a significant (P < 0.05) association between systolic BP and weight, age and HC only in girls (B ranges from −0.65 to 1.33). There was no significant association (P > 0.05) between systolic BP and all the variables (age, BMI, weight, height, WC, HC, WHR, triceps, subscapular and body fat %) (B ranges from −0.56 to 40.64) in boys. Moreover, there was a significant (P < 0.05) association in diastolic BP and subscapular among girls (B = 0.453).

Table 5 shows logistic regression showing the relationship between blood pressure and the physiological and anthropometric characteristics of Polokwane Private School children unadjusted and adjusted by age and gender. There was a nonsignificant (P > 0.05) association between systolic and diastolic BP and WHR when adjusted for age and gender (OR = 0.123 and 0.659, respectively). There was a significant (P < 0.05) association between systolic BP and WC unadjusted (OR = 1.125) and adjusted (OR = 1.097) for age and gender. There was also a significant (P < 0.05) association between systolic BP with body fat % unadjusted (OR = 1.131) and adjusted (OR = 1.128) for age and gender.

## 4. Discussion

The aim of this study was to determine the relationship between body composition and blood pressure among Polokwane Private School children. The current study found several significant correlations between both SBP and DBP with age, weight, height, BMI, WC, HC, WHR, tricep skinfold thickness, subscapular skinfold thickness and body fat % among Polokwane Private School children (girls and boys) aged 5 to 15 years. A study by Mushengezi and Chillo [18] found similar results, but also including significant correlation with fat mass. Another study by Gomwe et al. [2] also showed numerous anthropometric variables that were significantly correlated with SBP in children, including stature, weight, BMI, WC, triceps, subscapular, gluteal skinfold thicknesses and percentage body fat, excluding WHR. A study by Gaskin et al. [19] reported that there was an association between constantly increased blood pressure and increased body size and gender-specific body composition characteristics among adolescents. 

As indicated on the findings of the current study, among other anthropometric body composition variables, BMI and height were consistently significantly associated with SBP in correlation, linear regression and logistic regression. A study by Kuciene and Dulskiene [20] reported that body mass and stature in boys and body mass in girls were the most influential predictors of SBP. Furthermore, it was observed that age and body size in boys accounted for most of the SBP differences than percentage body fat [13,21]. This finding proposes that age and body size may also account for a greater proportion of SBP other than body fat. Moreover, the risk of having an increased BP increased with increasing levels of percentage body fat. Similar results were observed with different methods being used to measure the adiposity. Others used increased triceps skinfold thickness [22], while others used excess percentage body fat to examine the association between BP and adiposity in children and adolescents [23].

The prevalence of high SBP was 5.7% and 4.8% in girls and boys, respectively, while high DBP was 6.1% and 5.1% in girls and boys, respectively. The prevalence of overall hypertension was 5.3%. A similar study reported the prevalence of high SBP in girls of 5.6% and 4.8% for boys. The findings were similar to those when DBP was used [2]. In both studies, there is a similarity of girls having high BP, whether SBP or DBP. This can partly be accounted by girls having a significantly higher mean weight and BMI than boys which are both risk factors for high BP and other cardiovascular diseases. This is supported by Lloyd et al. [4], who further reported that increased weight is associated with a rise in hypertension, and this association can lead to atherosclerotic disease later in life. However, the overall prevalence of high blood pressure in this study was low. This can be because the children and adolescents in these schools are encouraged to take part in sport and most of their parents are educated, hence they could be making better choices in diet and activities for their children at home.

The limitations of this study are that the BP readings were recorded as the average of three measurements, which were taken after 5 minutes apart according to standard procedures, measurements were only taken on one occasion. Therefore, the possibility of bias cannot be excluded in the results. The current study did not have the resources to assess body composition using more direct indicators of body composition (i.e., body fat and lean fat mass) such as Bioelectrical Impedance, Hydrostatic Weighing and DEXA (Dual-Energy X-Ray Absorptiometry), among others. The strengths of the study were the large sample size and the different analytical methods used to determine the same association.

## 5. Conclusions

There was a significant association between body composition and blood pressure among Polokwane Private School children. There was an inconsistent association between blood pressure and waist-to-hip ratio. There was an overall low prevalence of high BP in the current study, however girls were more at risk than boys. Since the children are young and dependent on their parents/guardians, schools should regularly have educational programs to teach parents about healthy living (i.e., children must have a healthy weight/body mass index for their age, be physically active on a regular basis, eat a healthy diet that is low in salt, children must not smoke and drink alcohol, etc.) and the dangers associated with unhealthy habits. Therefore, the parents can make better choices and teach the children. This will lower the children’s risk of cardiovascular diseases in adulthood.

## Figures and Tables

**Table 1:** Physiological and anthropometric characteristics of Polokwane Private School children by gender.

	All (n = 1665)	Boys (n = 846)	Girls (n = 819)	P-Value
Characteristics	M (sd)	M (sd)	M (sd)	M (sd)
Age (years)	9.88 (2.12)	10.02 (2.15)	9.73 (2.08)	0.005 *
Weight (kg)	32.13 (10.28)	31.58 (9.14)	32.70 (11.31)	0.026 *
Height (cm)	139.27 (13.08)	139.17 (12.30)	139.38 (13.84)	0.754
BMI	16.20 (2.99)	16.01 (2.67)	16.39 (3.30)	0.010 *
WC (cm)	57.23 (6.51)	57.74 (5.90)	56.70 (7.05)	0.001 *
HC (cm)	70.58 (9.64)	68.70 (8.29)	72.53 (10.51)	0.000 *
WHR	0.82 (0.059)	0.85 (0.05)	0.79 (0.05)	0.000 *
Triceps (mm)	10.82 (4.68)	10.12 (4.73)	11.55 (4.51)	0.000 *
Subscapular (mm)	7.75 (4.20)	6.92 (3.75)	8.61 (4.46)	0.000 *
SBP (mmHg)	101.41 (13.81)	101.07 (13.19)	101.77 (14.42)	0.299
DBP (mmHg)	68.39 (10.60)	68.11 (10.59)	68.69 (10.61)	0.269
Body fat %	20.19 (5.84)	16.77 (4.81)	23.74 (4.55)	0.000 *

M(sd): mean (standard deviation); n, number of participants; BMI, body mass index; WC, waist circumference; HC, hip circumference; WHR, waist-to-hip ratio; SBP, systolic blood pressure; DBP, diastolic blood pressure. * P < 0.05

**Table 2:** Descriptive frequencies of systolic and diastolic blood pressure among Polokwane Private School children by gender (total n, boys = 846; girls = 819).

Blood Pressure	All	Males	Females
	n (%)	n (%)	n (%)
High SBP (mmHg)	88 (5.3)	41 (4.8)	47 (5.7)
High DBP (mmHg)	93 (5.6)	43 (5.1)	50 (6.1)
Hypertension	71 (4.3)	28 (3.3)	43 (5.3)

SBP, systolic blood pressure; DBP, diastolic blood pressure; n, number of participants.

**Table 3:** Pearson correlation between systolic and diastolic blood pressure and the physiological and anthropometric characteristics of Polokwane Private School children by gender.

	All		Boys		Girls	
	SBP	DBP	SBP	DBP	SBP	DBP
	r (P)	r (P)	r (P)	r (P)	r (P)	r (P)
Age (years)	0.481 **	0.285 **	0.475 **	0.244 **	0.542 **	0.308 **
Weight (kg)	0.574 **	0.398 **	0.556 **	0.372 **	0.593 **	0.335 **
Height (cm)	0.511 **	0.312 **	0.509 **	0.295 **	0.553 **	0.292 **
BMI	0.438 **	0.363 **	0.382 **	0.318 **	0.423 **	0.262 **
WC (cm)	0.484 **	0.365 **	0.470 **	0.326 **	0.477 **	0.271 **
HC (cm)	0.546 **	0.396 **	0.518 **	0.253 **	0.562 **	0.329 **
WHR	−0.264 **	−0.178 **	−0.245 **	−0.167 **	−0.360 **	−0.227 **
Triceps (mm)	0.330 **	0.299 **	−0.245 **	0.209 **	0.333 **	0.252 **
Subscapular (mm)	0.386 **	0.313 **	0.312 **	0.217 **	0.401 **	0.286 **
SBP (mmHg)	−	0.640 **	−	0.554 **	−	0.638 **
DBP (mmHg)	0.640 **	−	0.554 **	−	0.638 **	−
Body fat%	0.310 **	0.262 **	0.283 **	0.220 **	0.378 **	0.274 **

BMI, body mass index; WC, waist circumference; HC, hip circumference; WHR, waist to hip ratio; SBP, systolic blood pressure; DBP, diastolic blood pressure; ** P < 0.001.

**Table 4:** Linear regression showing the relationship between blood pressure and the physiological and anthropometric characteristics of Polokwane Private School children by gender.

	Boys			Girls		
	B	95% CI	P Value	B	95% CI	P Value
**Systolic blood pressure**						
Age (years)	0.66	−0.027–0.1338	0.060	1.33	0.514–2.140	0.001 *
Weight (kg)	0.18	−0.590–0.953	0.644	−0.65	−1.248–2.140	0.035 *
Height (cm)	0.11	−0.275–0.488	0.583	0.46	0.168–0.755	0.002 *
BMI	0.50	−0.246–2.239	0.576	1.82	0.546–3.088	0.005 *
WHR	40.64	−60.671–141.947	0.431	87.20	−10.178–184.581	0.079
WC (cm)	−0.56	−2.131–1.012	0.485	−1.01	−2.310–0.286	0.126
HC (cm)	0.83	−0.514–2.173	0.226	1.14	0.095–2.196	0.033 *
Triceps (mm)	−0.18	−0.750–0.383	0.526	0.14	−0.330–0.618	0.551
Subscapular (mm)	0.12	−0.385–0.619	0.647	0.06	−0.374–0.502	0.773
Body fat%	−0.13	−0.0750–0.490	0.681	−0.06	−0.633–0.509	0.831
**Diastolic blood pressure**						
Age (years)	−0.40	−0.99–0.187	0.181	0.947	0.266–1.634	0.007 *
Weight (kg)	−1.10	−0.77–0.56	0.765	0.019	−0.490–0.527	0.943
Height (cm)	0.20	−0.13–0.53	0.224	0.035	−0.213–0.283	0.784
BMI	1.48	−0.02–2.99	0.053 *	0.275	−0.800–1.350	0.616
WHR	−27.70	−114.98–59.57	0.533	75.79	−74.78–89.94	0.857
WC (cm)	0.47	−0.88–1.83	0.492	−0.285	−1.38–0.812	0.610
HC (cm)	−0.19	−1.35–0.96	0.492	0.166	−0.723–1.055	0.714
Triceps (mm)	0.39	−0.99–0.877	0.740	0.235	−0.166–0.637	0.250
Subscapular (mm)	−0.04	−0.47–0.39	0.118	0.453	0.082–0.083	0.017 *
Body fat%	−0.64	−1.17–0.106	0.859	−0.100	−0.583–0.384	0.686

BMI, body mass index; WC, waist circumference; HC, hip circumference; WHR, waist to hip ratio; SBP, systolic blood pressure; DBP, diastolic blood pressure; * P < 0.05.

**Table 5:** Logistic regression showing the relationship between blood pressure and the physiological and anthropometric characteristics of Polokwane Private School children unadjusted and adjusted by age and gender.

	Unadjusted			Adjusted for Age and Gender		
	OR	95%CI	P Value	OR	95%CI	P Value
**Systolic Blood Pressure**						
Weight (kg)	1.098	1.078–1.108	0.000 **	1.086	1.061–1.112	0.000 **
Height (cm)	1.079	1.059–1.100	0.000 **	1.065	1.034–1.097	0.000 **
BMI	1.250	1.186–1.318	0.000 **	1.198	1.134–1.265	0.000 **
WC (cm)	1.125	1.095–1.155	0.000 **	1.097	1.067–1.129	0.000 **
HC (cm)	1.106	1.084–1.128	0.000 **	1.096	1.069–1.123	0.000 **
WHR	0.000	0.000–0.011	0.000 **	0.123	0.001–26.351	0.444
Triceps (mm)	1.137	1.100–1.176	0.000 **	1.108	−1.071–1.148	0.000 **
Subscapular (mm)	1.132	1.097–1.169	0.000 **	1.106	1.069–1.144	0.000 **
Body fat%	1.131	1.093–1.170	0.000 **	1.128	1.085–1.172	0.000 **
**Diastolic blood pressure**						
Weight (kg)	1.090	1.170–1.109	0.000 **	1.087	1.062–1.112	0.000 **
Height (cm)	1.058	1.040–1.076	0.000 **	1.038	1.007–1.069	0.014 *
BMI	1.271	1.206–1.341	0.000 **	1.230	1.164–1.299	0.000 *
WC (cm)	1.125	1.097–1.155	0.000 **	1.106	1.076–1.138	0.000 **
HC (cm)	1.100	1.079–1.122	0.000 **	1.099	1.073–1.126	0.000 **
WHR	0.001	0.000–0.043	0.000 **	0.316	0.002–52.491	0.659
Triceps (mm)	1.153	1.116–1.191	0.000 **	1.130	1.093–1.169	0.000 **
Subscapular (mm)	1.144	1.108–1.180	0.000 **	1.122	1.086–1.160	0.000 **
Body fat%	1.129	1.091–1.167	0.000 **	1.130	1.089–1.174	0.000 **

OR, odds ratio; BMI, body mass index; WC, waist circumference; HC, hip circumference; WHR, waist to hip ratio; SBP, systolic blood pressure; DBP, diastolic blood pressure; * P < 0.05; ** P < 0.001.
